# Practical Points

**Published:** 1894-10-20

**Authors:** 


### PRACTICAL POINTS.

[Questions are invited for this column on any point in the administration of hospitals and asylums which may prove of difficulty or interest to our readers. All communications should be marked "Practical Points," addressed to the Editor of The Hospital, 428, Strand, W.C.]

*Teak Floors.—Is there any objection to the use of teak as a flooring for hospital wards? I have heard them objected to as noisy.—Matron.*

Teak floors are always regarded as excellent. We have never heard the objection of noise raised in connection with them. If the floors objected to are already laid down, the defect could be remedied by slag-wool plugging. There is no better non-conductor of sound. Polishing is the best treatment for the floor surface.

